# Marked under-diagnosis of Lambert-Eaton myasthenic syndrome in small cell lung cancer: an analysis of real-world claims data

**DOI:** 10.3389/fonc.2025.1650373

**Published:** 2025-10-17

**Authors:** Benjamin J. Drapkin, David J. Morrell, Regina Grebla, Guy Shechter, David E. Gerber

**Affiliations:** ^1^ Harold C. Simmons Comprehensive Cancer Center, University of Texas (UT) Southwestern Medical Center, Dallas, TX, United States; ^2^ Hamon Center for Therapeutic Oncology Research, University of Texas (UT) Southwestern Medical Center, Dallas, TX, United States; ^3^ Department of Internal Medicine (Division of Hematology-Oncology), University of Texas (UT) Southwestern Medical Center, Dallas, TX, United States; ^4^ Catalyst Pharmaceuticals, Inc., Coral Gables, FL, United States; ^5^ Northeast Epi, LLC, Bradford, NH, United States; ^6^ MedTech Analytics, LLC, Lexington, MA, United States; ^7^ O’Donnell School of Public Health, University of Texas (UT) Southwestern Medical Center, Dallas, TX, United States

**Keywords:** autoimmune, claims data, Lambert-Eaton myasthenic syndrome (LEMS), neurology, oncology, paraneoplastic, real-world, small cell lung cancer (SCLC)

## Abstract

**Background:**

Lambert-Eaton myasthenic syndrome (LEMS) is an autoimmune neurologic condition causing progressive muscle weakness that can occur as a paraneoplastic disorder, most commonly in patients with small cell lung cancer (SCLC). In limited prospective and retrospective studies, LEMS incidence in SCLC populations ranges 3-6%. Because LEMS may present a diagnostic challenge, we determined the prevalence of LEMS in a large, real-world, U.S.-based SCLC cohort.

**Materials and methods:**

We conducted a retrospective analysis of administrative data from Symphony Health’s PatientSource^®^, which represents over 300 million U.S. patients. In the primary analysis, we identified claims for LEMS (available starting in 2014) among patients with lung cancer claims between 2017 and 2022 who received etoposide and platinum-based chemotherapy (a validated approach to SCLC case identification).

**Results:**

Among 867,170 patients with lung cancer claims, 46,995 (5.4%) received platinum-etoposide-based therapy (putative SCLC cohort), of whom 77 (0.16%) had LEMS claims. In a subset of 8,513 patients with ≥12 months of claims preceding and following lung cancer diagnosis, 16 (0.19%) had LEMS claims. LEMS cases were more frequently diagnosed by neurologists (30%) than by oncologists (13%).

**Conclusions:**

In a large real-world cohort of patients with lung cancer, LEMS is diagnosed far less frequently than would be expected and rarely by oncologists. Because LEMS may convey substantial morbidity and specific LEMS treatments are available, further efforts to understand and address this discrepancy are warranted.

## 1 Introduction

Lambert-Eaton myasthenic syndrome (LEMS) is an autoimmune neurologic disorder that is most commonly identified as a paraneoplastic complication of small cell lung cancer (SCLC) (1). Unlike myasthenia gravis, a clinically similar disorder that presents with ptosis, diplopia, dysphagia, and dysarthria (2), the clinical hallmarks of LEMS are less distinctive. These include proximal muscle weakness, autonomic dysfunction, and areflexia, which may be misattributed to cancer, cancer-directed therapy, or go undetected (3). Proximal muscle weakness may be misinterpreted as general fatigue, a common symptom of SCLC and a common side-effect of chemotherapy (4). Similarly, symptoms of autonomic dysfunction, such as lightheadedness, early satiety, and constipation, may be misattributed to SCLC, chemotherapy, or palliative medications such as opioid analgesics. Loss of deep tendon reflexes is a key physical finding for LEMS diagnosis (5), but may not be assessed thoroughly outside of neurology practices (6, 7). Due to these challenges and the role of voltage gated calcium channel (VGCC) antibody and electromyography (EMG) testing in confirming diagnosis, practice guidelines recommend early involvement of neurologists in the evaluation of suspected LEMS cases (8). Given these considerations, the subtle presentation of LEMS in the confounding presence of SCLC may increase the risk of missed or delayed diagnosis.

Epidemiologic data on the diagnosis of LEMS among SCLC patients is limited. In prospective European SCLC cohorts, the prevalence of LEMS is approximately 3% (9–12). Retrospective U.S. studies report estimates ranging from 3.7% among patients with limited-stage SCLC to 5.9% in autopsy series (13, 14). Conversely, approximately half of patients with LEMS in European and U.S. cohort studies had SCLC, with most SCLC diagnoses occurring within 3 months of the LEMS diagnosis and presenting as limited stage disease (9–12, 14–19).

Importantly, none of these earlier studies included patient data after 2018, when immune checkpoint inhibitors became standard-of-care for first-line management of SCLC (20, 21). These inhibitors may exacerbate mild or sub-clinical paraneoplastic autoimmune disorders, with an unclear impact on LEMS diagnosis (22, 23). Because there are effective therapies for LEMS (e.g., amifampridine (24, 25)), missed diagnoses may result in substantial excess morbidity. We therefore evaluated the frequency and timing of LEMS diagnoses among contemporary lung cancer patients using real-world medical claims data. Because diagnostic coding systems do not distinguish between SCLC and non-small cell lung cancer (NSCLC), we used a previously established etoposide treatment-based algorithm to identify SCLC cases (26).

## 2 Materials and methods

### 2.1 Study design

We performed a retrospective analysis of de-identified patient-level data from Symphony Health’s PatientSource database. PatientSource data include longitudinal medical and pharmacy healthcare claims for over 300 million individuals across the United States (U.S.) with commercial and government (Medicare and Medicaid) insurance. These data have been used previously to study diagnostic and treatment patterns for a variety of diseases, including inflammatory bowel disease, osteoporosis, human immunodeficiency virus (HIV), multiple sclerosis, and narcolepsy (27–32). We used time-restricted database extracts for oncology (October 2017 to April 2022) and LEMS (March 2014 to July 2022) for this analysis. Because this study used fully de-identified, HIPAA-compliant claims data that contained no protected health information, the study did not constitute human subjects research as defined under 45 CFR 46. Therefore, the study did not require Institutional Review Board approval.

Using International Classification of Diseases, Ninth Revision, Clinical Modification (ICD-9-CM) codes 162.X (malignant neoplasm of the trachea, bronchus, and lung) excluding 162.0 (malignant neoplasm of the trachea) and ICD-10-CM codes C34.X (malignant neoplasm of the bronchus and lung), we identified patients with lung cancer in the oncology database extract based on the claims rule-out method (presence of ≥2 claims ≥30 days apart) (33). The first observed qualifying lung cancer claim during the study period served as the patient’s index date. We followed a similar approach to identifying LEMS cases [ICD-9-CM: 358.3 (LEMS), 358.30 (LEMS unspecified), 358.31 (LEMS in neoplastic disease), 358.39 (LEMS in other diseases classified elsewhere) and ICD-10-CM codes G70.80 (LEMS unspecified), G70.81 (LEMS in disease classified elsewhere), G73.1 (LEMS in neoplastic disease)] among patients with lung cancer.

We collected the following patient characteristics: age (on the index date), sex, insurance type (commercial, Medicare, Medicaid, and other), and geographic area (U.S. Census Bureau statistical region: Northeast, Midwest, South, and West). Among patients in the primary analysis, we recorded the specialty of the clinician associated with the index LEMS claim.

### 2.2 Data analysis

Because coding systems do not distinguish between SCLC and the more common NSCLC, we employed previously described and validated algorithms to identify SCLC cases (26, 34–36). Specifically, we estimated the period prevalence (and associated 95% CIs) of LEMS among patients with SCLC using three approaches:

For the primary analysis, we identified LEMS claims among a sample of patients with lung cancer claims between 2017 and 2022 who also received etoposide and platinum-based (carboplatin or cisplatin) chemotherapy. ICD-9-CM and ICD-10-CM codes do not distinguish between non-small cell lung cancer (NSCLC, approximately 85% of lung cancer cases in the U.S.) and SCLC (approximately 10-15% of lung cancer cases in the U.S.). Because this regimen represents the preferred first-line systemic therapy for both limited- and extensive-stage SCLC and is rarely employed in NSCLC, we used this treatment to distinguish SCLC from NSCLC, an approach taken in previous studies using administrative claims data (26, 34, 35).

We also analyzed LEMS claims in a subset of patients from the primary analytic cohort who had follow-up for ≥12 months of claims history before and after the index lung cancer diagnosis. Recognizing that this approach could skew findings toward longer-surviving patients, we also expected more patients in this subset to have a LEMS diagnosis because the median time interval between SCLC diagnosis and LEMS diagnosis exceeds 4 months (37, 38).

Third, because SCLC cannot be identified specifically, we determined the prevalence of LEMS among all patients with any lung cancer claims in the oncology dataset, with the expectation that only 10-15% of lung cancer claims in the dataset might be SCLC based on the relative frequency of SCLC versus NSCLC observed in the US population (39).

We also performed sensitivity analyses as follows: (1) We modified our claims-based approach to include LEMS diagnoses based on (a) a single LEMS claim and (b) ≥3 LEMS claims spanning ≥90 days. (2) Because etoposide and platinum-based therapy with concurrent chemoradiotherapy are occasionally used in Stage 3 NSCLC, as well as limited-stage SCLC, we searched for claims for radiotherapy within 30 days of a claim for etoposide or cisplatin or carboplatin among those with lung cancer claims with and without a LEMS diagnosis. (3) We also evaluated the receipt of etoposide without platinum-based therapy, as SCLC patients deemed unfit for doublet therapy may be treated with this approach (40).

We determined the time interval between the index SCLC diagnosis and LEMS diagnosis. To permit sufficient observation time to capture both diagnoses, for this analysis we used the LEMS dataset extract, which provided an additional 3 years of claims history (2014-2022) and included patients with ≥12 months of claims history in the oncology dataset. As in prior studies (15), we considered diagnoses of SCLC and LEMS to be concurrent if the first observed claims for each fell within 90 days of each other. The proportion of patients diagnosed with LEMS before or after their index SCLC and their median absolute times to diagnosis were compared.

Characteristics among patients in the study cohort with and without LEMS claims, and among patients with lung cancer claims with (primary sample) and without platinum-etoposide claims (other patients with lung cancer), were compared using two-sample t-tests or Wilcoxon rank sum tests for continuous variables and chi-square and Fisher’s exact tests for categorical variables.

## 3 Results

**Figure 1:**
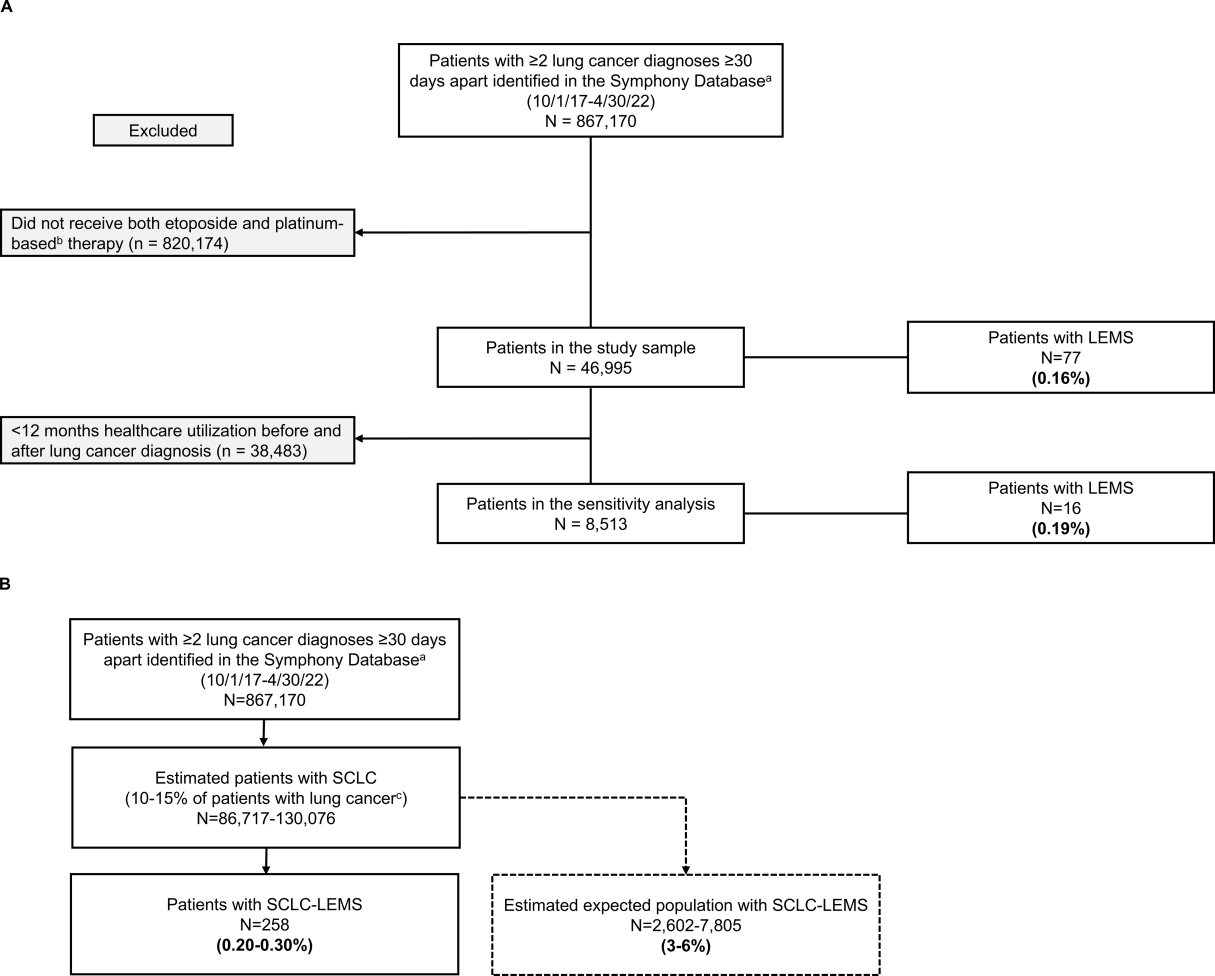
Estimated prevalence of LEMS in patients with SCLC in the PatientSource database according to two methodologies. **(A)** Using receipt of platinum-etoposide chemotherapy to identify presumed SCLC cases. **(B)** Applying SCLC prevalence estimates to identify presumed SCLC cases. LEMS, Lambert-Eaton Myasthenic Syndrome; N, number of patients in the population; SCLC, small cell lung cancer.

**Figure 1** displays two approaches used to estimate LEMS prevalence in SCLC: using receipt of platinum-etoposide chemotherapy to indicate presumed SCLC cases (**Figure 1A**) and applying SCLC prevalence estimates to indicate presumed SCLC cases (**Figure 1B**). Among 867,170 patients with eligible lung cancer claims between 2017 and 2022, 46,995 (5.4%) had claims for both etoposide and platinum-based therapy. These individuals were presumed to have SCLC and constituted the primary study sample. Seventy-seven patients in the primary analysis (0.16% [95% CI, 0.13%-0.20%]) had a claim for LEMS during the study period. In the subset of presumed SCLC patients with ≥12 months’ follow-up (n=8,513), 16 (0.19% [95% CI, 0.10%-0.28%]) had a LEMS claim.

Sensitivity analyses among patients in the primary study sample using a single LEMS claim, as well as ≥3 LEMS claims ≥90 days apart, yielded estimates of 0.25% and 0.12%, respectively. Based on epidemiologic estimates of SCLC occurring among 10-15% of patients with lung cancer (corresponding to n=86,717-130,076 of all patients with lung cancer claims in our dataset) in the U.S.), we calculated an expected LEMS prevalence of 0.20% (95% CI, 0.17%-0.22%) to 0.30% (95% CI, 0.26%-0.33%) in SCLC.

Provider specialty associated with the index LEMS claim among patients with presumed SCLC, according to timing of diagnoses, are presented in **Table 1**. The type of specialist associated with the index LEMS claim was examined according to the timing of diagnoses. Among 54 patients with information on provider specialty and ≥12 months claims history before their index lung cancer diagnosis, 22 (41%) had an associated neurology visit. All three patients whose first observed LEMS claim fell >90 days before their index lung cancer claim saw a neurologist, while 15 (39%) and 4 (33%) patients had a neurology visit associated with LEMS within 90 days or more than 90 days after the index lung cancer claim, respectively.

**Table 1:** Provider specialty associated with the index LEMS claim among patients with presumed^a,b^ SCLC, according to timing of diagnoses (N = 76).

	LEMS >90 days before SCLC (N = 3)	LEMS within 90 days of SCLC (N = 55)	LEMS >90 days after SCLC (N = 18)
Specialist associated with index LEMS claim, n (%)			
Emergency Medicine	0 (0)	1 (2)	0 (0)
Family Medicine	0 (0)	2 (4)	1 (6)
General Surgery	0 (0)	1 (2)	0 (0)
Hematology/Oncology	0 (0)	4 (7)	1 (6)
Infectious Diseases	0 (0)	0 (0)	1 (6)
Internal Medicine	0 (0)	5 (9)	3 (17)
Nephrology	0 (0)	1 (2)	0 (0)
Neurology	3 (100)	13 (24)	2 (11)
Neurology; Pathology, Anatomic/Clinical; Radiology	0 (0)	1 (2)	0 (0)
Neurophysiology, Clinical	0 (0)	1 (2)	2 (11)
Oncology Medical	0 (0)	3 (6)	1 (6)
Pathology, Anatomic/Clinical	0 (0)	1 (2)	0 (0)
Physical Medicine & Rehabilitation	0 (0)	3 (6)	0 (0)
Pulmonary Care Critical Medicine	0 (0)	1 (2)	0 (0)
Pulmonary Diseases	0 (0)	0 (0.0)	1 (6)
Radiology Oncology	0 (0)	1 (2)	0 (0)
Thoracic Surgery	0 (0)	1 (2)	0 (0)
Missing/Unknown	0 (0)	16 (29)	6 (33)

Among 76/77 treated SCLC-LEMS patients with ≥12 months claims history before lung cancer diagnosis. LEMS, Lambert-Eaton Myasthenic Syndrome.

^a^Lung cancer claims with ≥2 diagnoses ≥30 days apart.

^b^Patients received both etoposide and cisplatin or carboplatin chemotherapy.

Patient characteristics are presented in **Table 2**. Numerically, patients with SCLC with LEMS were younger than patients with SCLC without LEMS (mean 64.5 *vs*. 66.2 years) and more likely to be female, but neither difference reached statistical significance. Provider specialty information for the index LEMS claim was identified in 55 (71%) patients, with the most common specialties being neurology (42%), oncology (18%), and internal medicine (15%). Numerically, a higher proportion of patients with LEMS (46%) received concurrent radiotherapy relative to patients without LEMS (36%).

**Table 2:** Characteristics among patients in the primary sample with and without LEMS (N = 46,995).

	Cases without LEMS^a,b,c^ (N = 46,918)	Cases with LEMS^a,b,c^ (N = 77)	*P* value^d^
Age, years, mean ± SD	66.3 ± 38.1	64.5 ± 7.0	0.69
Female, n (%)	24,217 (51.6)	42 (54.5)	0.61
Insurance coverage, n (%)^e^			
Commercial	24,416 (52.0)	48 (62.3)	0.18
Medicare	15,444 (32.9)	16 (20.8)	
Medicaid	3,077 (6.6)	4 (5.2)	
Missing/unknown	5,841 (12.4)	9 (11.7)	
Other	850 (1.8)	0 (0)	
Census Region, n (%)			
Northeast	7,278 (15.5)	16 (20.8)	0.77
Midwest	12,179 (26.0)	20 (26.0)	
South	21,039 (44.8)	33 (42.9)	
West	5,937 (12.7)	8 (10.4)	
Unknown	278 (0.6)	0 (0)	

LEMS, Lambert-Eaton Myasthenic Syndrome; N, number of patients in the population; SD, standard deviation.

^a^Lung cancer claims with ≥2 diagnoses ≥30 days apart.

^b^Patients received both etoposide and cisplatin or carboplatin chemotherapy.

^c^Assessed on the index date.

^d^Statistical comparisons were performed using two-sample t-tests or Wilcoxon rank sum tests for continuous variables and chi-square and Fisher’s exact tests for categorical variables. A *P* value < 0.05 was considered statistically significant.

^e^Totals sum >100% as patients may have had >1 type of insurance during the study period.

Characteristics of patients in the primary sample with presumed SCLC (N = 46,995) versus all other patients with lung cancer claims (presumed NSCLC; N = 820,175) using the various approaches are presented in **Table 3**. Patients with presumed SCLC were younger (66.3 *vs*. 68.3 years; *P* < 0.001), were more likely located in Southern and Midwestern Census regions, and had minor, but statistically significant, differences in the distribution of insurance type compared to all other patients with lung cancer claims. Among all other patients with lung cancer, 1,990 (0.2%) received etoposide without platinum-based therapy.

**Table 3:** Characteristics of lung cancer patients with SCLC and other lung cancer cases (N = 867,170).

	Presumed SCLC cases^a,b^ N=46,995	Other lung cancer cases^a^ N=820,175	*P* value^c^
Age, years, mean ± SD^d^	66.3 ± 38.1	68.3 ± 36.6	<0.001
Female, n (%)	24,259 (51.6)	421,551 (51.4)	0.35
Insurance coverage, n (%)^e^			
Commercial	24,464 (52.1)	431,552 (52.6)	<0.001
Medicare	15,460 (32.9)	287,482 (35.1)	
Medicaid	3,081 (6.6)	43,521 (5.3)	
Missing/unknown	5,850 (12.4)	79,605 (9.7)	
Other	850 (1.8)	13,354 (1.6)	
U.S. Census Region, n (%)			
Northeast	7,294 (15.5)	162,894 (19.9)	<0.001
Midwest	12,199 (26.0)	188,706 (23.0)	
South	21,072 (44.8)	340,567 (41.5)	
West	5,945 (12.7)	115,490 (14.1)	
Unknown	278 (0.6)	8,253 (1.0)	

LEMS, Lambert-Eaton Myasthenic Syndrome; N, number of patients in the population; SCLC, small-cell lung cancer; SD, standard deviation.

^a^Based on ≥2 diagnoses ≥30 days apart.

^b^Patients received both etoposide and cisplatin or carboplatin chemotherapy.

^c^Statistical comparisons were performed using two-sample t-tests or Wilcoxon rank sum tests for continuous variables and chi-square and Fisher’s exact tests for categorical variables. A *P* value < 0.05 was considered statistically significant.

^d^Assessed on the index date.

^e^Totals sum >100% as patients may have had >1 type of insurance during the study period.

Characteristics of 1,836 patients in the LEMS data set with and without eligible lung cancer claims are presented in **Table 4**. Among them, 390 (21.2%) had lung cancer claims and were significantly older (65.7 *vs* 58.7 years; *P* < 0.001) than patients with LEMS without lung cancer claims.

**Table 4:** Characteristics of patients with LEMS with and without lung cancer claims^a^ (N = 1,836).

	LEMS without lung cancer N=1,446	LEMS with lung cancer^a^ N=390	*P* value^b^
Age, years, mean ± SD^c^	58.7 ± 15.2	65.7 ± 7.6	<0.001
Female, n (%)	840 (58.1)	212 (54.4)	0.19
Insurance coverage, n (%)^d^			
Commercial	986 (65.2)	246 (63.1)	0.16
Medicare	355 (24.6)	100 (25.6)	
Medicaid	76 (5.3)	28 (7.2)	
Missing/unknown	86 (5.9)	33 (8.5)	
Other	30 (2.1)	10 (2.6)	
Census Region, n (%)			
Northeast	281 (19.4)	79 (20.3)	0.79
Midwest	347 (24.0)	103 (26.4)	
South	557 (38.5)	149 (38.2)	
West	243 (16.8)	58 (14.9)	
Unknown	2 (0.1)	0 (0)	

LEMS, Lambert-Eaton Myasthenic Syndrome; N, number of patients in the population; SCLC, small-cell lung cancer; SD, standard deviation.

^a^Based on ≥2 diagnoses ≥30 days apart.

^b^Statistical comparisons were performed using two-sample t-tests or Wilcoxon rank sum tests for continuous variables and chi-square and Fisher’s exact tests for categorical variables. A *P* value < 0.05 was considered statistically significant.

^c^Assessed on the index date.

^d^Totals sum >100% as patients may have had >1 type of insurance during the study period.

**Figure 2:**
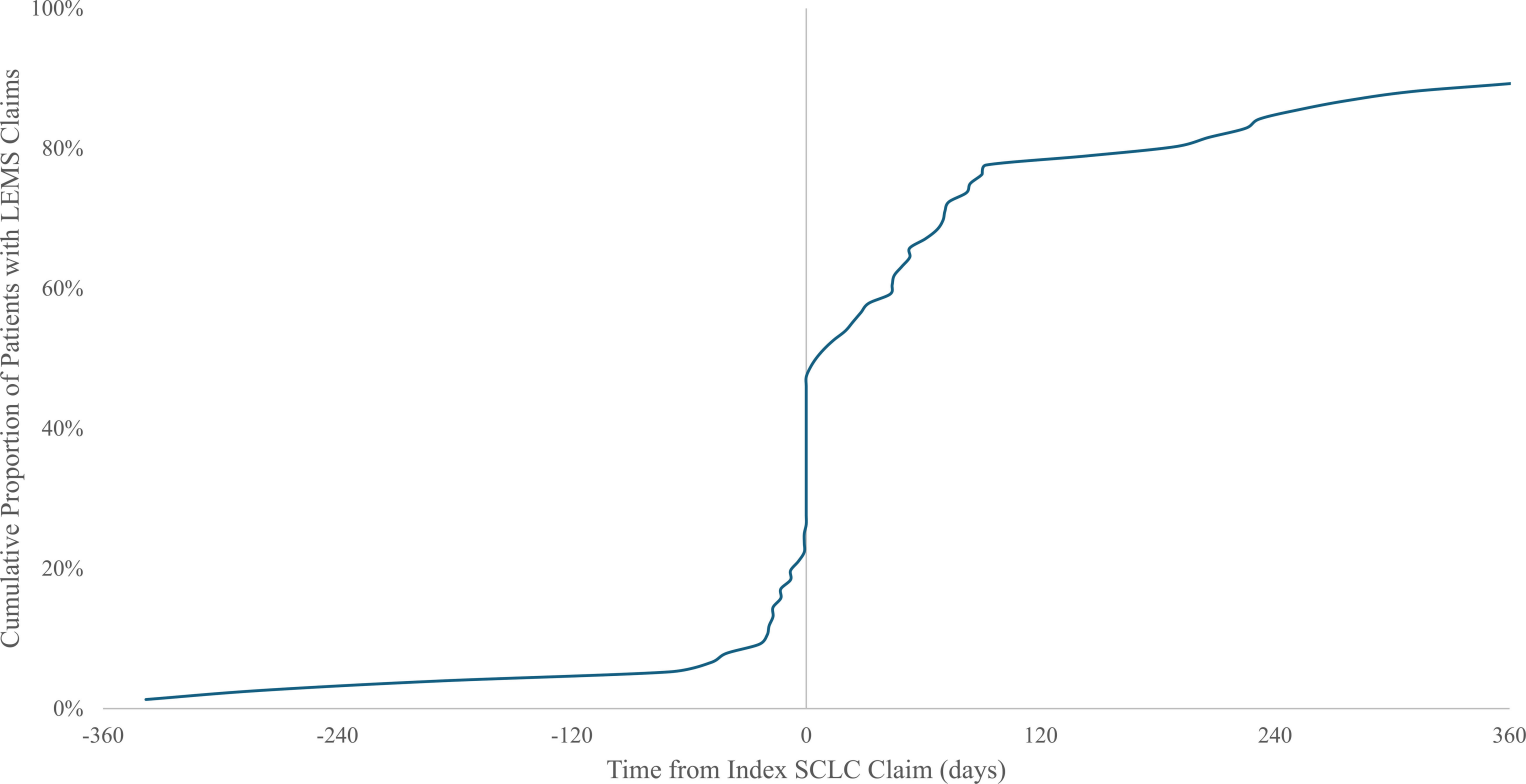
Time between diagnosis of small-cell lung cancer (SCLC) and diagnosis of Lambert-Eaton myasthenic syndrome (LEMS). Cohort includes 76 patients with treated presumed SCLC and diagnosed LEMS with ≥12 months claims history before lung cancer diagnosis. LEMS, Lambert-Eaton Myasthenic Syndrome; SCLC, small-cell lung cancer.

The timing of LEMS and SCLC diagnoses among 76 patients with SCLC and LEMS in the primary sample with ≥12 months of claims history is illustrated in **Figure 2**. Overall, the median time between diagnoses was 37 (interquartile range [IQR] 1-155) days. Nineteen patients (25%) had LEMS claims that preceded lung cancer claims. Among them, most (84%) patients had a diagnosis of lung cancer within 90 days (median 17 [IQR 8-45] days). Seventeen patients (22%) had initial LEMS and lung cancer diagnoses on the same day, while 40 patients (53%) had a first LEMS claim after the index lung cancer claim. Among these patients, the median interval between diagnoses was 83 (IQR 45-286) days, and 18 patients (45%) had LEMS diagnoses >90 days after their index lung cancer claim.

Because immunotherapy use could hypothetically increase the occurrence and/or severity of autoimmune processes, we compared LEMS prevalence before and after the standardization of immune checkpoint inhibitor use in SCLC treatment. Although the anti-PD1 antibody nivolumab was FDA approved as third-line therapy for SCLC in August 2018, we did not select this cut-off because (1) systemic therapy beyond second-line is rarely administered in SCLC due to clinical deterioration and disease burden at that point (41); (2) later lines of therapy would generally be employed only several months after diagnosis and therefore not affect earlier clinical course (41); and (3) approval of this regimen was withdrawn in 2021 after phase 3 trials did not show an overall survival benefit compared to chemotherapy or placebo in relapsed SCLC (42, 43). Instead, we used March 2019 (approval of first-line platinum-etoposide + atezolizumab [anti-PDL1 antibody], a regimen that remains in widespread use today) as the cut-off. We identified 46 LEMS cases in the ICI period and 31 cases in the pre-ICI period, corresponding to prevalence rates of 0.18% and 0.15%, respectively (*P* = 0.49).

## 4 Discussion

This real-world analysis suggests that LEMS is diagnosed in fewer than 0.3% of patients with presumed SCLC in the U.S., a rate less than one-tenth that reported in prospective studies of patients with SCLC (9–12). While we cannot determine whether this discrepancy represents an overestimate in prospective studies or an underestimate in this contemporary real-world analysis, given the non-specific nature of LEMS symptoms and the preponderance of studies reporting rates of 3% or greater, it seems likely that LEMS remains underdiagnosed in real-world settings.

Our sensitivity and subset analyses may provide further insight into LEMS diagnoses. Among patients who received etoposide monotherapy, who generally have poor performance status precluding platinum administration, we identified no LEMS cases. Whether undiagnosed LEMS contributed to this state for some cases is unknown. We also observed that presumed SCLC patients with LEMS were more likely to receive concurrent chemoradiotherapy than were SCLC patients without LEMS, suggesting that LEMS cases were more likely to have limited-stage disease, consistent with prior retrospective and prospective studies demonstrating comparatively favorable survival outcomes for SCLC patients with LEMS (18, 19).

Among patients diagnosed with LEMS after SCLC, the median time to LEMS diagnosis was four months, with intervals exceeding one year in some patients. Patients with SCLC-LEMS have a median survival of 18 months (19). Because diagnostic challenges prolong the time to LEMS diagnosis in both tumor- and non-tumor LEMS patients (1), it becomes clear that LEMS diagnoses may be missed altogether. Indeed, fewer than half of patients with available information in our study were diagnosed with LEMS by a neurologist. When LEMS diagnoses followed SCLC diagnoses, the diagnosis was even less likely to be made by a neurologist relative to LEMS diagnoses preceding SCLC diagnosis, reflecting the opportunities for oncologists to recognize and confirm the condition.

In an earlier study, patients with SCLC and LEMS were younger than patients with SCLC without LEMS (median 63 versus 66 years old) (9). We similarly found that patients with presumed SCLC and LEMS were younger on average than presumed SCLC patients without LEMS. We also observed, consistent with previous studies, that patients with LEMS without lung cancer claims were significantly younger than those with presumed SCLC (12). However, in contrast to the male predominance in prior reports of SCLC-LEMS (10, 12, 44), more than half of the putative SCLC-LEMS patients in the present study were female. One possible explanation is the inherent difference between prospective studies, in which surveys, physical exams, and serologic assessment may reveal LEMS, while a real-world claims database population may require patient report of physical symptoms—which women are more likely to do than men (45).

In the present analysis, fewer than one-fourth of LEMS patients had lung cancer diagnoses, considerably lower than the approximately 50% SCLC prevalence among patients with LEMS intensively screened for SCLC (15, 38). Factors beyond screening may account for our findings. Decreases in the incidence of SCLC parallel lower rates of smoking in the U.S. and may account for the lower observed prevalence of SCLC among patients with LEMS in our study (46). Similarly, one-third of patients with LEMS had SCLC in a recent nationwide survey in Japan, a country in which similar decreases in smoking occurred during the study period (47). In addition, women in the US are less likely than men to report current smoking (48) and may contribute to the lower SCLC prevalence observed in our study, where over half of patients with LEMS were female.

A major strength of this analysis is the use of real-world claims data, which provided information on the largest U.S. cohort of SCLC-LEMS patients to date. However, the primary limitation of this analysis reflects the inherent challenges of capturing SCLC diagnoses using claims data, as lung cancer diagnostic (ICD) codes are not histology-specific. Accordingly, our strategy of using prototypical treatment regimens as a surrogate marker of these cases may misclassify cases. That said, this etoposide treatment-based approach to SCLC case identification has been used previously and validated against electronic health record (EHR) histology information (26, 34–36). Nevertheless, we investigated potential sources of misclassification, including locally advanced NSCLC, for which platinum-etoposide chemotherapy is occasionally administered with concurrent thoracic radiotherapy. In our primary sample, about 35% of patients received concurrent radiotherapy. Notably, LEMS was diagnosed more frequently in this subset than in patients who did not receive radiotherapy. This finding may reflect the expected greater likelihood of limited stage SCLC among radiotherapy-treated patients and might suggest relatively little dilution with NSCLC cases. The exclusion of cases treated with etoposide monotherapy had minimal effect on our estimates, as only 0.2% of patients in the dataset received this regimen and none had a LEMS diagnosis.

Nevertheless, the results of this analysis should be interpreted with caution as the identification of both SCLC and LEMS in the database relied upon accurate coding and algorithms that could be validated against EHR. As a result, we report the diagnosed prevalence of LEMS among a population of patients with presumed SCLC and acknowledge that the true prevalence of SCLC-LEMS may not be fully captured. The requirement of healthcare utilization claims post-index diagnosis in the sensitivity analysis may introduce survival bias, as patients with SCLC may not survive long enough to be diagnosed with LEMS. Additionally, the observational datasets used for this study did not include patient race-ethnicity or smoking history, important information for understanding real-world LEMS patterns and broader epidemiologic trends. Cash-paying and uninsured patients were not included in the database, limiting the generalizability of our findings and estimated prevalence estimates to insured individuals. Lastly, we were unable to distinguish incident from prevalent LEMS diagnoses in the analysis due to left truncation of the dataset.

Despite these considerations, because the prevalence of LEMS across analyses in the present analysis fell under 0.3% of cases—at most, one-tenth the rate expected in a SCLC population—it seems highly likely that LEMS is, indeed, substantially underdiagnosed. In addition, more than 50% of LEMS claims followed the presumed SCLC claims with a substantial delay. If this reflects LEMS diagnosis that occurs after the start of first-line therapy, then by current guidelines LEMS diagnosis would be expected to follow treatment with immune checkpoint inhibitors for almost all patients (20, 21). Given the hypothetical risk that these agents could increase the prevalence and severity of LEMS (although we did not observe such an association in the present study) and other paraneoplastic neurologic syndromes (49, 50), recent changes in SCLC management have increased the importance of early LEMS diagnosis and management.

In conclusion, in a large, representative, and contemporary real-world population, over 90% of SCLC-associated LEMS cases appear to go undetected. This marked underdiagnosis occurs despite availability of effective LEMS treatment that can improve patients’ quality of life. To address this care gap, efforts to understand and mitigate the reasons for this profound and persistent under-recognition are clearly needed.

## Data Availability

The datasets presented in this article are not readily available because the data that support the findings of this study are available from ICON plc (Symphony Health). Restrictions apply to the availability of these data, which were used under license for this study. Requests to access the datasets should be directed to david.gerber@utsouthwestern.edu.
